# From Traditional to Digital: Examining the Transformation of Sociocultural Barriers and Facilitators in Healthcare Technology Adoption

**DOI:** 10.21203/rs.3.rs-6995030/v1

**Published:** 2025-07-28

**Authors:** Jacqueline B. Duong, Natalia Simo Fiallo, Sierra N. Walters, Kayla E. Carta, Grace A. Jumonville, Alyssa S. Carrasco, Daniela N. Romero, Ishita P. Khurd, Matthew W. Ahle, Jonathan S. Comer, Stacy L. Frazier, Theodora Chaspari, Shri Narayanan, Adela C. Timmons

**Affiliations:** The University of Texas at Austin; Florida International University; The University of Texas at Austin; The University of Texas at Austin; The University of Texas at Austin; The University of Texas at Austin; The University of Texas at Austin; The University of Texas at Austin; Colliga Apps Corporation; Florida International University; Florida International University; University of Colorado; University of Southern California; The University of Texas at Austin

**Keywords:** digital health tools, health disparities, digital divide, mixed methods

## Abstract

As digital health technologies (DHTs) reshape healthcare delivery, it remains unclear how they transform access barriers and facilitators, particularly for historically marginalized populations. This convergent mixed-methods study examined how diverse caregivers experience traditional versus digital healthcare. Thematic analysis of interviews and correlational analyses of health and digital literacy revealed how key access factors evolve with digital adoption. Financial constraints persisted across both contexts but shifted in form, while digital settings introduced new challenges, including relational disconnect, technology access gaps, and data privacy concerns. Facilitators also differed; traditional care emphasized interpersonal and cultural supports, whereas DHTs enabled flexibility and personalization. Social identity shaped these experiences, with identity-linked barriers more frequently referenced in traditional care. This suggests disparities may become less visible, but not less present, in digital environments, highlighting the need for continued vigilance. Findings support inclusive, equity-centered design strategies to ensure digital innovations promote, rather than obscure, equitable healthcare access.

## Introduction

Despite extensive research and policy initiatives, health disparities remain a persistent challenge in the United States^1,2^. These disparities are rooted in unequal access to and use of healthcare services, disproportionately impacting marginalized communities^3^. The rapid growth of digital health technologies (DHTs), including telehealth, mobile health apps, and remote monitoring, has the potential to bridge these gaps by increasing healthcare access, enhancing patient engagement, and enabling personalized care^4–6^. However, the extent to which DHTs mitigate or reproduce existing barriers, particularly for structurally marginalized groups, remains unclear^7^. This mixed-methods study examines how barriers and facilitators compare across traditional and digital healthcare contexts and how these dynamics intersect with social identities, with the goal of informing more equitable digital health design and implementation.

Health disparities, defined as preventable differences in health outcomes across population groups, are shaped by complex social determinants of health, including socioeconomic status, geographic location, educational attainment, and racial or ethnic identity^2,8,9^. Social factors influence health through several key pathways: unequal access to quality healthcare^10,11^, differences in community resources and environmental conditions^12,13^, systemic barriers such as healthcare discrimination^14,15^, and language and cultural barriers that hinder communication between patients and providers^16^. The COVID-19 pandemic both illuminated and exacerbated these existing inequities, with marginalized communities experiencing disproportionately higher infection and mortality rates alongside reduced access to essential care^17,18^. Simultaneously, the pandemic catalyzed rapid DHT adoption, creating a critical moment to examine whether these innovations might mitigate or inadvertently reinforce existing disparities^19,20^. Importantly, early evidence from the COVID-19 pandemic period suggests that while telehealth became more widespread, it failed to significantly expand access for underserved populations, with usage still skewed toward higher-income, urban, and privately insured patients^7^.

DHTs show considerable promise for reducing health disparities by overcoming traditional barriers to care access. These tools can eliminate geographic constraints, reduce costs, provide 24/7 accessibility, and offer culturally tailored interventions^21,22^. However, the *digital divide*, defined as systematic differences in resources and capabilities needed to access and effectively use digital technologies, poses significant barriers, especially for marginalized and underrepresented groups^23,24^. This divide manifests in multiple dimensions, including physical access to technology, digital literacy, and meaningful use of digital resources for health improvement. For example, while smartphone access is relatively consistent across racial groups, Black and Hispanic adults are less likely than White adults to own laptops or have home broadband access^25,26^. These gaps limit participation in telehealth and other digital services, particularly in low-resource households and rural areas^27,28^. In some cases, digital tools may offer greater convenience for those already accessing care, rather than expanding access to new or marginalized users^29^.

Understanding what facilitates access to healthcare is as important as identifying its barriers, especially as healthcare delivery increasingly incorporates digital tools. In traditional healthcare settings, facilitators such as trusted providers, family support networks, and culturally grounded services have been shown to ease access and promote engagement, particularly for communities historically marginalized in the healthcare system^30,31^. In digital contexts, emerging evidence highlights new facilitators, including culturally tailored content, digital literacy support, and user-centered design^24,32,33^. Community-based digital interventions and peer networks have also been found to promote adoption and engagement among underserved populations^32,34^. DHTs may reduce traditional access barriers through increased flexibility, asynchronous communication, and customizable interfaces that accommodate a range of linguistic, cognitive, and physical needs^21,35^. Additionally, digital platforms may reduce barriers related to provider mistrust or facility-related stigma for some populations, with randomized trials showing improved treatment engagement among minoritized families receiving telehealth compared to office-based care^36^. This advantage may be particularly pronounced for families with young children, where telehealth enables treatment in natural home settings during a critical developmental period when children are transitioning to formal schooling (Sanchez et al., 2024). Together, these facilitators illustrate how healthcare support systems are evolving alongside shifts in delivery models, prompting new questions about how facilitation operates across both traditional and digital settings.

When transitioning from traditional to digital healthcare contexts, there is a widespread assumption that DHTs will automatically address existing barriers.. Although digital health innovations may successfully overcome some traditional barriers (e.g., geographic distance, scheduling constraints), they may simultaneously introducing new challenges (e.g., technology access limitations, digital literacy requirements, or privacy concerns in small dwellings or public settings), particularly for populations already facing technological accessibility barriers^37^. Similarly, the facilitators that help patients navigate traditional healthcare systems may not translate directly or effectively to digital contexts. Some populations may lose valuable support mechanisms when care shifts to digital platforms. Before developing and implementing effective digital health equity strategies, we need rigorous research examining how the landscape of healthcare barriers and facilitators translates across these contexts, particularly for historically marginalized communities who may experience compounded disadvantages.

It is also important to consider how healthcare barriers and facilitators intersect with social identities. According to intersectionality theory^38,39^, people’s experiences of discrimination are shaped by the combination of their social identities, like race, gender, income, and disability. In healthcare contexts, barriers and facilitators may manifest differently across various populations based on identity^40,41^. For example, challenges may include a lack of funds for devices, difficulty understanding technology due to age, and language barriers that hinder navigation of the interface. These considerations may be especially complex for caregivers managing both their own healthcare needs and those of school-age children, who face compounded demands during this developmental transition. These factors may differentially shape how individuals engage with traditional versus digital care, underscoring the importance of designing inclusive health interventions that equitably address the needs of historically underserved populations.

This study investigates three key questions: (1) How do DHTs reduce or introduce barriers to healthcare compared to traditional settings? (2) How do DHTs modify or introduce new facilitators for healthcare engagement? and (3) How are these barriers and facilitators related to social identities across both healthcare modalities? We began with qualitative thematic analysis to identify key barriers and facilitators in both traditional and digital healthcare contexts. A comparative analysis then examined how these themes evolved—whether they persisted, changed in character, or newly emerged—across modalities. Quantitative survey data on health literacy, eHealth literacy, technology use, and social identity were analyzed using descriptive statistics and correlation analyses to identify overall patterns across the sample. To integrate findings, we examined correlations between the frequency of qualitative themes and participants’ quantitative scores, identifying how reported experiences related to specific literacy, technology, and attitudinal variables. Finally, we examined how social identities shaped healthcare engagement by analyzing qualitative co-occurrence patterns between identity mentions and thematic codes, as well as quantitative correlations between identity variables, qualitative codes, and survey measures. This integrative approach deepens our understanding of how barriers and facilitators operate across both traditional and digital healthcare domains, helping to inform the development of more equitable, identity-responsive health solutions.

## Method

### Study Design

We employed a convergent mixed-methods approach to collect and analyze qualitative and quantitative data^42^ simultaneously. Semistructured interviews explored participants’ lived experiences with healthcare and digital technologies, while structured surveys provided additional context about health literacy, technology use, and privacy concerns. Both data types were collected concurrently and analyzed separately, with the results integrated during the interpretation phase to identify points of convergence and divergence^43^. Findings were triangulated from the combined data sources, with joint displays via correlational heatmaps utilized to visually integrate quantitative and qualitative results. The COnsolidated criteria for REporting Qualitative research (COREQ) checklist was used for reporting purposes^44^.

### Participants

Forty-eight caregivers, drawn from a larger study examining family mental health (*N* = 56), opted for in-depth qualitative interviews exploring their experiences with healthcare and DHTs (*M*age = 41.49, *SD* = 6.56, *range* = 26–56). One participant was excluded due to technical issues that prevented the collection of an adequate audio recording. For the qualitative component, the sample size aligns with established guidelines for achieving thematic saturation in interview-based research, typically with 20–30 interviews^42,45^. Although modest for quantitative analyses, the sample was intentionally selected to enable in-depth, intensive interviews. This design also permitted exploratory quantitative analyses, which yielded preliminary insights and highlighted emerging patterns that enriched and contextualized the qualitative findings. Given this sample size, interpretive emphasis was placed on effect sizes rather than statistical significance alone when examining quantitative patterns.

To better understand healthcare disparities among caregivers from diverse backgrounds, we established eligibility criteria focused on populations historically underserved in healthcare research and most likely to face such disparities. Caregivers of children aged 6–9 were included based on the design of the larger parent study, which focused on family and child mental health during early to middle childhood. This developmental window marks the start of formal schooling, introducing new academic and social demands that can influence children’s mental health trajectories and increase caregiver stress^46^. To ensure the sample reflected families navigating meaningful contextual or mental health challenges, participants had to meet at least one of the following criteria: identifying as a member of an underrepresented racial-ethnic group (93.5% of sample met this criterion), having a child with elevated mental health symptoms defined as scoring at or above the 70th percentile on at least one SDQ difficulty subscale; ^47^, or reporting household income at or below the 33rd percentile of their county, adjusted for family size (65.2% of sample met this criterion). These inclusion criteria were chosen to capture families experiencing structural, socioeconomic, or psychological factors that may shape parenting and engagement with child mental health services, without restricting the sample to a single clinical domain or dimension of adversity. English or Spanish language proficiency was also required to support meaningful participation and promote linguistic diversity in the sample. All inclusion criteria were guided by the American Psychological Association’s standards for inclusivity and bias-free language^48^. These inclusion criteria were designed to ensure representation of individuals most affected by structural healthcare inequities, aligning with the study’s intersectionality framework by examining how overlapping social identities shape healthcare experiences^38,39^.

### Procedure

Data collection occurred during the COVID-19 pandemic (2020–2023), a period that heightened existing healthcare disparities^49^ and accelerated the adoption of DHTs^50,51^. Participants were recruited through community flyers, referrals, research centers, clinics, and social media. Interested families completed a contact form, followed by an eligibility screening based on income, racial/ethnic background, and child mental health symptoms. Eligible participants signed consent/assent forms and completed questionnaires during a main study visit not relevant to the current study, as well as additional surveys on health literacy, eHealth literacy, technology usage, and privacy concerns for the present project. All participants then completed a semi-structured interview that explored their experiences with healthcare and digital technologies. Interviews and questionnaires were linked at the individual level for integrated analysis. Participants were compensated with $40.All procedures adhered to the ethical standards of the Declaration of Helsinki and were approved by the University IRB (reference number IRB-20–011). Interview recordings and questionnaire data were de-identified and encrypted. All study data, including consent forms, were stored in a secure database.

### Qualitative Data Collection

Semi-structured interviews lasting 45 to 50 minutes were conducted via Zoom video conference. The interview protocol was developed to explore participants’ experiences with traditional and digital healthcare, focusing on barriers, facilitators, and cultural influences on healthcare engagement. The semi-structured format allowed interviewers to ask follow-up probes to explore participants’ social identities and intersections with healthcare experiences. The discussion covered various topics, including current experiences within the healthcare system, attitudes towards and usage of DHT, and barriers and facilitators that impact healthcare access. The interview protocol was designed with branching logic to accommodate the varied experiences of participants. If participants indicated no current health concerns in response to initial questions, interviewers shifted focus to explore their general experiences with healthcare systems, preventive care practices, and attitudes toward digital health technologies.

The data collection team consisted of JD, NF, MS, PZ, ZB, AV, and JL, all of whom were female except for ZB, and were trained in either qualitative interviewing or analysis. JD was a doctoral student, NF was a a project coordinator, and MS, PZ, ZB, AV, and JL were research assistants. All have a bachelor’s degree except for JL. NF is Spanish-speaking. Only one interviewer (JD) was present for English interviews, while two researchers (JD and NF) conducted Spanish interviews to ensure accuracy in translation and understanding. A reflexivity statement is provided in Supplemental Materials (Text 1). Participants were informed about the research objectives and the team’s interest in understanding caregivers’ experiences with healthcare systems and digital health technologies. All interviews were audio and video recorded with participant consent for transcription purposes. Field notes were written by interviewers after each session and used to supplement transcription accuracy and provide contextual data during the coding and thematic analysis phases.

### Qualitative Analysis

English interviews (*n* = 40) were transcribed verbatim by research staff. Spanish interviews (*n* = 7) were professionally translated and transcribed into English to ensure consistency across the dataset for coding purposes. Thematic analysis was conducted using MAXQDA 2022^52^, following both inductive and deductive approaches. Initial coding was largely inductive, allowing salient patterns and themes to emerge directly from the data, while final theme development was guided by a deductive framework aligned with the study’s central research questions. To ensure analytic rigor, we implemented systematic coding procedures, team-based consensus building, and inter-coder agreement assessment. The analytic process began with team members engaging in data familiarization, re-reading transcripts, and recording initial impressions. During the open coding phase, six team members, led by JD, independently coded a randomly selected subset of interviews, focusing on domains including healthcare barriers and facilitators, digital technology experiences, cultural and identity-related influences, and technology adoption patterns. Identity-related codes were also used to explore how social background shaped healthcare and DHT engagement (see Table 4 for the full code list).

Codes were iteratively reviewed and refined through team meetings, during which coders compared interpretations, consolidated codes, and developed preliminary themes. All interviews were double coded to enhance trustworthiness, with discrepancies resolved via consensus. To integrate coders’ work, second-coder files were merged using MAXQDA’s Teamwork Import feature with the “use outer segment boundaries” option, preserving interpretive context and metadata. Of the 47 double-coded transcripts, 38 had sufficient segment overlap to calculate percent agreement and Cohen’s Kappa, which ranged from 0.72 to 0.92, indicating substantial to almost perfect agreement^53^. For the remaining 9 transcripts, IRR could not be computed due to export issues; however, all were resolved through team consensus (see Supplemental Materials, Table 1). This partial IRR strategy reflects best practices in applied thematic analysis, balancing rigor and feasibility in large-scale qualitative research^54,55^.

To align the analysis with our research objectives, we organized coding around four guiding questions: (1) What are the traditional barriers to healthcare? (2) What are the traditional facilitators to healthcare? (3) What barriers are introduced or sustained by DHTs? and (4) What facilitators are introduced by DHTs? After extracting the themes, we conducted a comparative analysis of the thematic structure to examine how barriers and facilitators shifted across contexts. Themes were categorized based on whether they persisted, changed, or newly emerged in the digital healthcare setting. This comparative approach allowed us to identify patterns of continuity and transformation, illuminating how participants’ experiences translated—or did not translate—between traditional and digital modes of care.

### Quantitative Data Collection

To complement the qualitative interviews, participants completed several validated scales to assess health literacy, eHealth literacy, technology use, and privacy concerns.

#### Health Literacy.

The Health Literacy Questionnaire HLQ; ^56^, a 44-item self-report measure, was employed to assess health literacy across nine domains: feeling understood and supported by healthcare providers, having sufficient information to manage health, actively managing health, social support for health, appraisal of health information, ability to actively engage with healthcare providers, navigating the healthcare system, ability to find good health information, and understanding health information well enough to know what to do. Sample items include “I feel I have good information about health” and “I have enough information to help me deal with my health problems.” Items are scored on a 4-point scale (1 = *strongly disagree* to 4 = *strongly agree*) for the first five domains or a 5-point scale (1 = *cannot do or always difficult* to 5 = *always easy*) for the remaining four domains. Each domain is scored independently by summing the relevant items, with possible scores ranging from 4 to 16 for the first three domains, 5 to 20 for domains 4 and 5, and 5 to 25 for domains 6 to 9. Higher scores indicate greater health literacy in that specific area. The HLQ has demonstrated strong construct validity, reliability, and acceptability across diverse populations, with reliability estimates ranging from 0.77 to 0.90 and a well-supported nine-factor structure^56^. In the present sample, Cronbach’s alpha ranged from 0.69 to 0.93 across the nine domains.

#### eHealth Literacy.

The eHealth Literacy Scale eHEALS; ^57^ is an 8-item tool to assess individuals’ ability to find, understand, evaluate, and apply health information from electronic resources. Sample items include “I know how to find helpful health resources on the Internet” and “I can tell high-quality health resources from low-quality health resources on the Internet.” Items are scored on a 5-point Likert scale (1 = *strongly disagree* to 5 = *strongly agree*), with total scores ranging from 8 to 40. Higher scores indicate greater eHealth literacy. The eHEALS has demonstrated strong internal consistency across diverse populations, with Cronbach’s α ranging from 0.88 to 0.97^58^, and has been validated in multiple contexts and languages^59,60^. In the present sample, the Cronbach’s alpha was 0.91.

#### Technology Use and Comfort.

The Technological Ease and Computer-based Habits Inventory TECHI; ^61^ assessed general technology use, comfort, and familiarity. This 17-item measure was developed based on existing technology adoption literature and assesses participants’ general technological proficiency by measuring the frequency of use and comfort with various digital technologies. Sample items include “I use a smartphone on a daily basis” and “People close to me would refer to me as tech savvy.” Items are scored on a 6-point scale (0 = *strongly disagree* to 5 = *strongly agree*) with total scores ranging from 0 to 85. The TECHI yields a total score and two subscale scores (Extent of Use and Technological Competency and Patience). For this study, we used the total score to assess overall technological proficiency, which aligned with our research questions examining general comfort and capability with technology. Higher scores indicate greater technological proficiency. In the present sample, Cronbach’s alpha was 0.81 for the total score.

#### Privacy and Security Concerns.

Finally, participants’ attitudes and concerns regarding mobile privacy and security were assessed using the Mobile Privacy and Security Concerns Scale^62^, a 3-item measure examining concerns about mobile health technology use. Items include “How concerned are you about privacy and security when sending or receiving messages on your mobile phone device?”, “How concerned are you about privacy and security from the apps you use on your mobile phone device?”, and “You limit the types of messages you send or receive on your mobile phone or device because you are worried about privacy and security.” The first two items are scored on a 4-point Likert scale (1 = *extremely concerned* to 4 = *not concerned at all*), while the third item is scored on a 5-point scale (1 = *strongly disagree* to 5 = *strongly agree*). For consistent interpretation, we reverse-coded the third item so that lower scores indicated greater privacy concerns across all items. The scale demonstrated good internal consistency in our sample (Cronbach’s α = .76). A composite privacy concerns score was created by summing all three items (possible range: 3–13), with lower scores indicating greater privacy concerns.

### Quantitative Analysis

Descriptive statistics were calculated for all quantitative variables. Given the sample size and potential non-normality of the data, Spearman’s rank-order correlations were used to examine interrelationships among key quantitative measures, including domains of health literacy, eHealth literacy, technological proficiency, and privacy concerns. These analyses provided insight into how different competencies and concerns clustered within the sample.

### Integration of Qualitative and Quantitative Analysis

We employed a multi-method integration strategy to examine convergence and divergence between qualitative and quantitative findings. First, we used data triangulation to compare emergent qualitative themes with patterns observed in the quantitative dataset. To assess how participants’ reported experiences aligned with their measured competencies, we then conducted Spearman’s rank-order correlations between thematic code frequencies and quantitative scores (e.g., HLQ, eHEALS, TECHI). This analytic approach enabled us to explore how individual differences in health literacy and technological proficiency related to the specific barriers and facilitators participants described in their narratives.

### Social Identity Intersections

To understand how intersecting identities shape healthcare experiences, we analyzed both qualitative and quantitative data through the lens of intersectionality theory, which emphasizes how multiple social identities (e.g., race, gender, socioeconomic status) intersect to influence individuals’ lived experiences of privilege and oppression^38,39^. Our analysis used two complementary approaches. First, we examined co-occurrence patterns in the qualitative data using MAXQDA’s code relations browser to explore how identity codes (e.g., race/ethnicity, income, education, sexual orientation) were linked with reported barriers and facilitators to healthcare access. This approach captured how participants themselves described the relevance of their identities in shaping their experiences. Second, we conducted Spearman’s rank-order correlations between participants’ social identity variables and both thematic coding frequencies and quantitative measures (e.g., HLQ, eHEALS, TECHI). These analyses enabled us to detect more subtle, statistically measurable patterns in how identity-based disparities were reflected in survey-reported competencies and qualitative themes.

## Results

### Demographic Information

The final sample comprised 47 female caregivers, including 18 monolingual English speakers (38.3%), 5 monolingual Spanish speakers (10.6%), 19 English-Spanish bilingual speakers (40.4%), 4 speakers of English or Spanish plus other languages (8.5%), and 1 multilingual speaker (2.1%). The sample was racially/ethnically diverse: Hispanic/Latino/a (36.2%), Black/African American (29.8%), Non-Hispanic White/Caucasian (14.9%), Multi-race (10.6%), Other (6.4%), and American Indian/Alaska Native (2.1%). Most participants were employed full-time (57.4%) with low to moderate income levels (80.85%). See Table 1 for more detailed demographic information.

### Qualitative Analysis

Thematic analysis revealed 18 distinct themes organized across four domains: Traditional Healthcare Barriers (five themes), Digital Health Technology Barriers (four themes), Traditional Healthcare Facilitators (four themes), and Digital Healthcare Facilitators (five themes). Table 2 presents an overview of the identified themes, along with their corresponding frequency counts.

### Traditional Healthcare Barriers

Five distinct barriers in traditional healthcare settings emerged: Financial Access Constraints, Structural System Limitations, Provider Bias and Discrimination, Cultural Belief Barriers, and Pandemic-Related Disruptions.

Financial Access Constraints, endorsed by 89% of participants, captured how personal and family financial circumstances directly affected healthcare access and decision-making, including difficult trade-offs between healthcare needs and other essential expenses. Participants frequently described delaying or avoiding care due to costs, struggling with insurance limitations, and experiencing stress related to medical expenses. As one participant explained: “*Sometimes I think about like, oh I should really go to a therapist you know, but… God they’re so expensive… like I could put that money towards like different things* [P51, age 39, Black, high income].” This testimony illustrates how financial constraints can create hierarchies of need, in which healthcare, particularly mental healthcare, is often deprioritized, even when individuals recognize its potential value. These financial barriers affected participants across income levels, with 96% of low-income participants (*n* = 23/24), 85% of middle-income participants (*n* = 11/13), and 78% of high-income participants (*n* = 7/9) reporting cost-related concerns, suggesting that cost considerations influence healthcare decisions regardless of financial resources.

Structural System Limitations (endorsed by 79% of participants) captured organizational impediments within the healthcare system that create or perpetuate access disparities distinct from individual financial constraints. These barriers included organizational inefficiencies, such as fragmented care coordination, administrative complexities like referral requirements and prior authorizations, and systemic workforce shortages that led to provider unavailability. These challenges often manifested as difficulty securing consistent, high-quality care. One participant shared:

It’s very frustrating to try to find a PCP [primary care provider]… I waited a month and a half, but… one thing that I think a lot of people struggle with is being able to make appointments to see doctors in a timely manner… All of my friends talk about having these problems all the time

[P59, age 34, Hispanic White, middle income].

This reflection highlights how institutional barriers, such as provider shortages and inefficient scheduling systems, can limit access to care even for those with sufficient financial resources, pointing to systemic issues embedded within the healthcare infrastructure itself.

Provider Bias and Discrimination (endorsed by 74% of participants) captured participants’ encounters with inequitable treatment in healthcare settings. Participants described systemic patterns of being dismissed, subjected to inadequate testing, or receiving lower-quality care—experiences disproportionately affecting marginalized groups. These concerns were often deeply entwined with racial and ethnic identity, amplifying barriers to trust and access. As one participant explained:

When it comes to Black-African American women,…[providers]…don’t go as far as the extent [to] helping them…my son goes to the doctor, he sees the shot…and he starts crying. “Oh, what’s wrong with him? He has a behavior problem?” No, he don’t like doctors

[P64, age 42, Black woman, low income].

This participant described a moment in which her child’s distress was pathologized rather than understood. Moments like these erode trust and reinforce feelings of being misunderstood and mistreated by the healthcare system.

Cultural Belief Barriers were endorsed by most participants (94%), making it the most endorsed traditional healthcare barrier. Cultural Belief Barriers described how cultural norms and family dynamics influenced the recognition and discussion of health conditions. This theme captured the complex interplay between cultural beliefs, communication patterns, and healthcare engagement. Participants described how cultural and regional traditions shaped communication about health concerns, often limiting open discussion of certain conditions. As one participant explained: “*Because my parents were from South Carolina, you didn’t go around talking about stuff; you kept everything to yourself, and that’s just the way it was, so I’ve suppressed a lot of stuff* [P35, age 56, non-Hispanic multiracial, middle income].” This participant explicitly connected her tendency to withhold health information to cultural practices she learned from her parents, demonstrating how cultural context shapes individual health behaviors and illustrates how regional cultural norms around health communication can be transmitted intergenerationally, creating patterns of health information suppression that may delay or prevent healthcare seeking.

Lastly, 60% of participants mentioned pandemic-related disruptions, highlighting how COVID-19 intensified existing healthcare disparities through a complex interplay between increased stressors and reduced access to care. Participants described how the pandemic simultaneously limited healthcare availability while heightening health needs and eliminating crucial support resources, such as childcare, compounding challenges. As one participant explained:

I’ve dealt with depression on and off my entire life and I’ve got that under control, but this is very different… it’s physically… I just feel like in the last year it’s like, my mental health has started to physically affect my body… I wasn’t speaking to [my counselor] a lot because I just couldn’t because I was with my kid and I didn’t have time and I didn’t have childcare

[P57, age 43, non-Hispanic white, high income].

This account illustrated the pandemic’s circular effect on health—increased stress led to worsening mental health with physical manifestations, while simultaneously creating barriers (lack of childcare, time constraints) that prevented consistent access to care. The prevalence of these experiences suggests the pandemic created unique structural challenges rather than simply exacerbating existing healthcare barriers.

### Digital Health Technology Barriers

Four distinct themes emerged as barriers to utilizing DHTs: relational connection loss, digital literacy and access gaps, data security concerns, and value-cost assessment.

Relational Connection Loss emerged as a barrier to DHT use, mentioned by 72% of participants. This theme centered on concerns about diminished interpersonal elements of healthcare when delivered through digital platforms. Participants described how virtual platforms could not replicate the nuanced dynamics of face-to-face healthcare encounters, potentially compromising care quality and therapeutic relationships. As one participant stated: “*I prefer mental health in person, especially for [child’s name]. For me, I could deal with it, but I prefer the human connection* [P31, age 37, Hispanic, middle income].” This sentiment reflected a multifaceted concern about digital healthcare delivery, including difficulties in establishing therapeutic rapport, challenges in maintaining engagement without in-person accountability, and limitations in remote assessment, particularly for children and those with physical conditions. The prevalence of this concern across demographic categories suggests that preserving human connection represents a core challenge for digital health implementation, regardless of technological proficiency or healthcare literacy. This pattern held across income levels (67% of low-income, 62% of middle-income, and 100% of high-income participants), education levels (40% of those with high school education or less, 64% of those with some college, and 82% of those with bachelor’s degrees or higher), age groups (68% of those under 40, 77% of those 40–50, and 50% of those over 50), and racial/ethnic groups (73% of Hispanic/Latino, 60% of Black/African American, and 100% of White participants)

Digital Literacy and Access Gaps, mentioned by 45% of participants, captured the dual challenges of technical proficiency and technology access that can impede effective utilization of digital health resources. These barriers showed interesting patterns across educational levels, with 57% of participants with some college education experiencing these gaps, compared to 40% of those with low education and 39% of those with higher education, suggesting that digital literacy challenges may not follow traditional educational hierarchies. The challenges often extended to family members who become de facto technical support:

My mom… trying to get her on whatever platform, like Zoom…I have to walk her through like every step of logging on… I think for people that are not tech savvy, it has been a little bit of a barrier for them because they don’t know how to access… the portals

[P49, age 45, Black, middle income].

This quote illustrates how digital literacy barriers can create additional burden on family members and potentially compromise healthcare independence.

Data Security Concerns were endorsed by 36% of participants, encompassing apprehensions about data protection and digital security that influence users’ willingness to engage with DHTs. “*The only other concern would be hackers, but I’m sure they’ll find a way around it* [P60, age 30, Black, low income].” This statement conveys a sense of resignation, acknowledging data breaches as an unavoidable feature of digital life, which may lead users to approach digital health tools with caution or reluctance.

Lastly, Value-Cost Assessment, mentioned by 28% of participants, reflected both financial constraints and attitudes about the perceived value of investing in digital health tools. Unlike traditional healthcare barriers where financial factors primarily affected access to care, in the digital context, these factors influenced both the ability to access technology and willingness to invest in digital health solutions: “*I tried them but to really get the meat from [the app], you have to pay, and I don’t believe in paying for apps. Some [apps] are really expensive. I would be into them if they were covered by insurance* [P2, age, 43, Hispanic White, low income].” This reflection illustrates how the intersection of cost barriers and value perceptions shapes engagement with digital health tools, presenting a unique challenge distinct from traditional healthcare cost barriers. Although traditional healthcare costs were typically viewed as essential, digital health tools were subject to additional scrutiny, with participants questioning whether their benefits justified the expense.

### Traditional Healthcare Facilitators

Although participants faced significant barriers in traditional healthcare settings, they also identified crucial facilitating factors that aided their healthcare navigation. Our analysis revealed four key traditional healthcare facilitators: supportive relationship networks, healthcare knowledge brokers, cultural resource integration, and non-clinical digital resources.

Supportive Relationship Networks emerged as the most prevalent traditional facilitator (endorsed by 94% of participants), underscoring the essential role of close personal ties in navigating healthcare. These networks, comprising family, friends, and partners, offered more than just emotional support. They functioned as relational safety nets, offering encouragement, helping interpret medical information, and guiding participants through logistical hurdles in the healthcare system. The support did not necessarily depend on formal medical expertise but on trust, availability, and lived experience. As one participant described: “*I do have a good network. My older sister’s a social worker…and will be able to tell me, ‘Hey listen, go visit this doctor*’ [P47, age 39, Hispanic, low income].” This quote illustrates how emotional and practical support often overlap in everyday caregiving relationships, reinforcing participants’ ability to access and act on healthcare needs.

Healthcare Knowledge Brokers, endorsed by 77% of participants, referred to a specific subset of support relationships—those in which the person providing help had formal training or insider knowledge of the medical system. These individuals, often family members or friends with clinical or administrative expertise, served as informal healthcare consultants. Their guidance often shaped how participants interpreted symptoms, navigated diagnoses, or sought specialized care. For example: “*My aunt is a psychiatrist. She did all her testing on me when I was a young kid, so it’s always been part of my life—you need help, you ask for it* [P13, age 33, Multiracial, middle income].” These brokers were especially powerful facilitators because they bridged the gap between layperson and professional healthcare systems, providing tailored guidance grounded in medical knowledge.

Additionally, Cultural Resource Integration, mentioned by 55% of participants, reflected the crucial role of cultural traditions, spiritual practices, and community values in shaping healthcare decisions and treatment preferences. For many, these resources served not only as complements to formal healthcare systems, but as vital sources of emotional support, caregiving, and healing. Participants described how religious communities, in particular, often filled gaps left by clinical services, providing childcare, emotional reassurance, and a sense of belonging. As one participant explained:

I have people that go to my church, that help me with my family, that give my children love even, even in times when I would say I don’t give my children the love that they deserve. I can go to church and then they’ll get love from you know people in my church, so I, I, I love it…I have a huge village of people that’s willing to help me and I love it, it’s just, I have to open up my mouth and ask for help

[P56, age 37, Black, Low income].

This narrative highlights how faith communities operate as extended support systems, offering resources and care beyond those typically available in healthcare settings. It also illustrates how cultural values shape not only what types of support are available, but also the norms surrounding help-seeking behavior, such as the emphasis on communal responsibility and the personal challenge of asking for assistance and the behavioral norms around accessing them.

Lastly, Non-Clinical Digital Resources, endorsed by 23% of participants, reflected the use of general online platforms, such as social media, forums, and search engines, as tools for navigating traditional healthcare systems. These platforms did not provide direct care but served as crucial intermediaries for locating providers, researching treatments, and connecting with others facing similar challenges. As one participant described: “*I was going online, I’m not even exaggerating when I say this, I’m buying insulin off of other people online and I- there’s a big network right now on Facebook actually of people who buy even used insulin from other people* [P24, age 47, Hispanic White, high income].” This account highlights how, in the absence of accessible formal resources, individuals turn to informal digital networks to meet pressing medical needs. These platforms function not only as information hubs but also as makeshift support systems, filling gaps when traditional channels fall short.

### Digital Healthcare Facilitators

Five distinct themes emerged as facilitators of DHT use, reflecting patterns in participants’ narratives that diverged from traditional facilitators in both form and their relationship to social identity. These included: accessibility and flexibility, personalized health management, financial incentivization, engagement mechanisms, and identity protection.

Accessibility and flexibility emerged as the most frequently cited digital facilitator (endorsed by 96% of participants), with many highlighting how digital health tools, particularly telehealth, help reduce traditional barriers related to time, distance, and scheduling.

I didn’t like telehealth before, but now…I like it because I can sit in my living room at 7:15, do my kids doctor appointment and then they can go to school… For a check in, it’s just made my life so much easier now… My perspective has completely changed about technology over the last year

[P41, age 35, Black, middle income].

This example illustrates how digital tools can meaningfully alleviate logistical challenges in traditional care by offering greater scheduling flexibility and convenience.

Personalized Health Management was discussed by 57% of participants, capturing how digital tools enable customized health monitoring and intervention. Unlike traditional healthcare’s episodic nature, digital tools offered continuous, personalized health tracking and feedback tailored to individual needs and preferences. Participants described how these tools could be adapted to simultaneously track various aspects of their health journey. As one participant explained: “*Basically it gives you ideas about how to think, mindfulness… do the breath app with the Apple Watch… going on the treadmill or outside for a walk or any type of exercise you can link it…it’s all on there* [P01, Non-Hispanic White, Low income].” This participant’s description shows how digital tools can provide personalized guidance across multiple domains, from mental wellbeing through mindfulness to physical activity across various exercise modalities, creating an integrated health experience tailored to individual preferences and behaviors. This level of customization enables users to receive continuous support and feedback tailored to their specific health goals and daily activities, a stark contrast to the standardized approaches often found in traditional healthcare settings.

Financial incentivization, mentioned by 23% of participants, reflected the perceived economic advantages of digital health tools, particularly when linked to insurance benefits or reward systems. In contrast to traditional healthcare’s often opaque and unpredictable costs, digital tools were seen as more transparent and financially motivating. As one participant explained: *Yeah, I use Apple Fitness and it’s called United Healthcare Emotion. It’s like an app for my health insurance and it connects to my watch, and it set goals through it and it has like a savings bank … you rack up like money in this bank. So, I can see the rewards of different goals that I meet… When I see the money sign, I’m like oh yeah, I’m up to $60* [P18, age 29, Hispanic, middle income].

This example highlights how integrating digital health tools with financial incentives can reinforce health behaviors by making progress visible and materially rewarding.

Engagement Mechanisms endorsed by 40% of participants encompassed specific features that promote consistent healthcare engagement through gamification, goal setting, and performance tracking. DHTs transformed routine health activities into more engaging experiences by applying behavioral science strategies such as reinforcement, goal setting, and socially driven motivation. Features like streak tracking, rewards, and leaderboards tapped into users’ motivation for achievement and competition, encouraging engagement. As one participant explained:

I think I’m naturally competitive so it’s like I want to get to my steps in my goal, and once I’m like- even if I get close to it, then it’s like I can’t stop. But if I like like today I’ll have like 1000 like I won’t even try because like I’m nowhere near it. But if I was at four or five then I’d be like trying more

[P57, age 43, Non-Hispanic White, High income].

This quote illustrates how gamified elements can spark engagement by activating a threshold of perceived attainability—users are more likely to persist when goals feel within reach. These patterns underscore the need for thoughtfully designed feedback to sustain user motivation over time.

Lastly, while endorsed by only a small subset of participants (6%), Identity Protection emerged as a meaningful counterpoint to concerns about relational connection loss. This theme highlighted how digital interfaces can facilitate the disclosure of sensitive health information by reducing face-to-face anxiety and perceived judgment. As one participant explained:

I think the digital health tools might help because…you’re not actually sitting there face to face with someone because I was just telling my counselor it’s a lot easier to talking to him, because he doesn’t know who I am, he’s never seen me, all of our calls are just phone calls, and so I feel more at ease talking to him about things

[P35, age 56, non-Hispanic multiracial, middle income].

This perspective, though less common, reveals the multifaceted nature of human connection in healthcare contexts, where physical distance can sometimes enable greater psychological openness. The low prevalence yet clear articulation of this theme suggests digital tools may offer unique advantages for addressing stigmatized health conditions or for individuals who experience anxiety in traditional healthcare interactions.

### Comparing Traditional and Digital Healthcare Barriers and Facilitators

#### Traditional and Digital Barriers Comparison.

**Figure 1:**
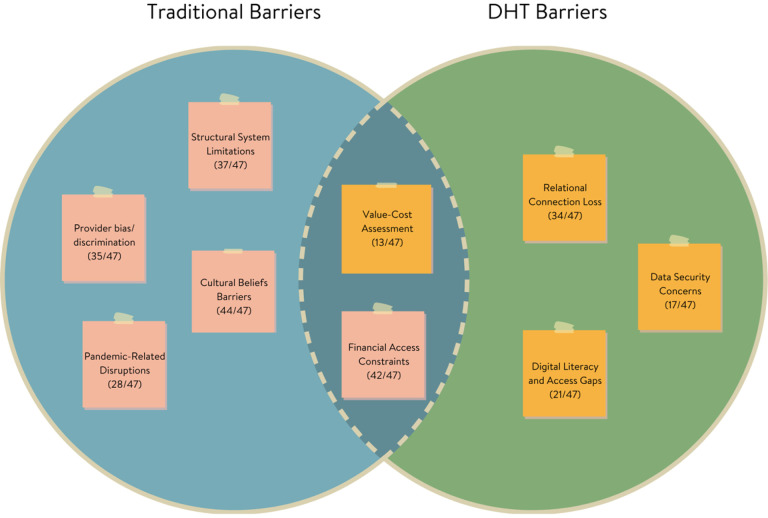
Comparison of barriers across traditional and digital healthcare contexts **Note.** Visual representation showing how barriers transform from traditional to digital healthcare settings, with overlapping and distinct elements highlighted

Healthcare barriers shifted in form, prevalence, or salience as participants considered traditional versus digital healthcare contexts (Fig. 1). Of the eight total barrier themes identified, one transformed across settings, four were specific to traditional care and did not surface in digital contexts, and three emerged in digital healthcare. Financial Access Constraints was the only barrier that persisted across both traditional and digital contexts, though it decreased in endorsement and shifted in its expression. In traditional settings (endorsed by 89% of participants), it referred to out-of-pocket costs and insurance gaps, whereas in digital contexts (28%), it focused more on technology costs and subscription fees, as well as their perceived value relative to their benefits.

By contrast, four barriers commonly cited in traditional care were not reported in digital contexts. These included structural system limitations (79%), such as difficulty scheduling or navigating clinic bureaucracy; provider bias and discrimination (74%); pandemic-related disruptions (60%); and cultural belief barriers (94%), which encompassed stigma, mistrust of formal systems, and preference for culturally grounded care. Although these themes were absent as standalone barriers in the digital context, some cultural influences may have been indirectly reflected in digital themes, especially through participants’ hesitancy to pay for app-based care or their discomfort with unfamiliar digital models. This overlap indicates that certain traditional barriers may not disappear but instead become folded into the framing of new digital concerns.

At the same time, three new barriers emerged exclusively in digital contexts. Relational connection loss (72%) captured how the absence of face-to-face interaction in telehealth and app-based care diminished emotional rapport, connection, or trust. Digital literacy and access gaps (45%) surfaced as a key challenge, reflecting disparities in technological comfort, device access, and internet connectivity. Finally, data security concerns (36%) reflected participants’ fears about privacy, surveillance, and digital vulnerability, which were unique to the digital format.

#### Traditional and Digital Facilitator Comparison.

**Figure 2:**
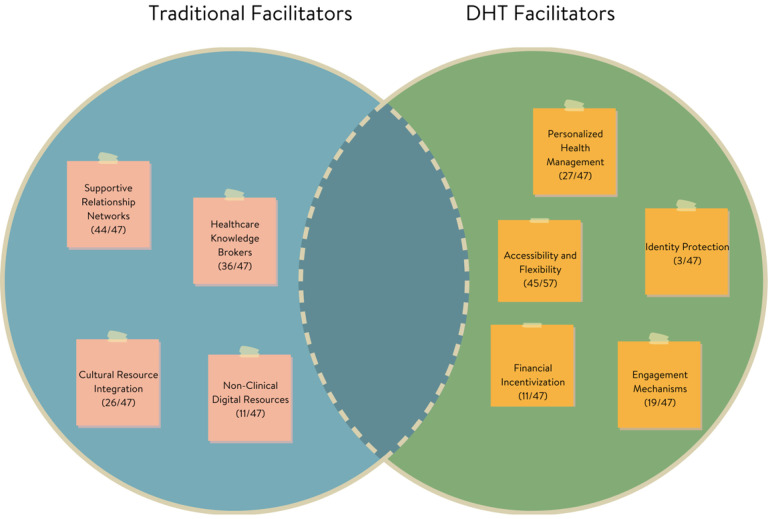
Comparison of facilitators across traditional and digital healthcare contexts **Note.** Visual representation illustrating the distinct nature of facilitators in traditional versus digital healthcare settings, showing minimal overlap between domains

Facilitators of healthcare access and engagement also shifted across traditional and digital health contexts (Fig. 2). Of the nine total facilitator themes, none were shared across both contexts in an identical form, though one showed conceptual overlap. Four were unique to traditional care, and five emerged specifically in digital contexts, reflecting how evolving healthcare modalities give rise to new support mechanisms while phasing out others.

In traditional healthcare, participants emphasized interpersonal and community-based facilitators. These included Supportive Relationship Networks (94%), such as family or friends who offered emotional support and help navigating care; Healthcare Knowledge Brokers (77%), referring to support figures with medical expertise; Cultural Resource Integration (55%), including faith-based and cultural community supports; and Non-Clinical Digital Resources (23%), such as using social media or forums to locate care. These facilitators underscored the relational and contextual nature of traditional healthcare access, particularly for communities that rely on informal systems of trust and guidance.

Digital contexts introduced an entirely different set of facilitators shaped by the nature of technology. Accessibility and Flexibility (96%) was the most endorsed digital facilitator, reflecting how digital health tools removed barriers of time, transportation, and scheduling. Participants also valued Personalized Health Management (57%), including goal-setting and self-monitoring features; Engagement Mechanisms (40%), such as gamification and feedback loops; Financial Incentivization (23%), which linked health behaviors to rewards or insurance benefits; and Identity Protection (6%), with some participants feeling more comfortable disclosing health issues in private, digital spaces.

### Quantitative Analysis

**Figure 3:**
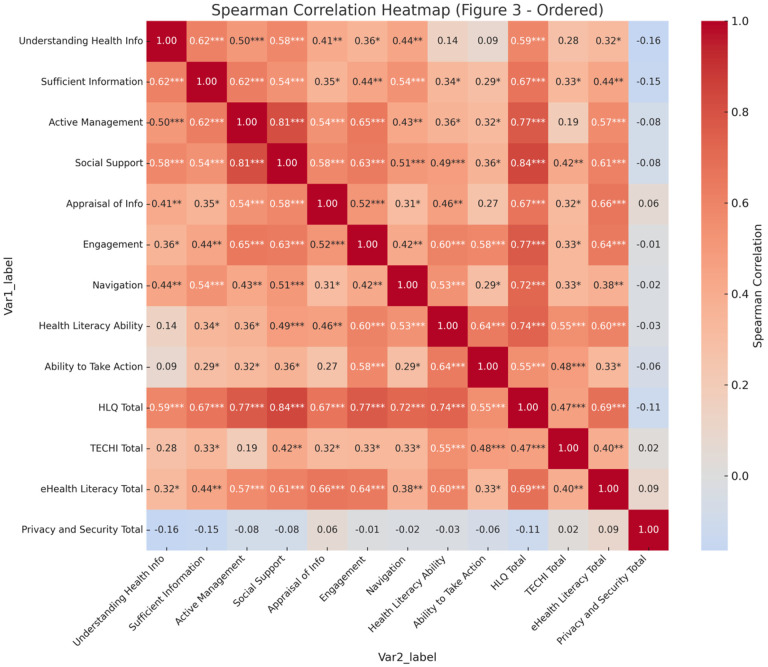
Spearman correlation matrix across health literacy, technology experience, eHealth literacy, and privacy and security variables **Note.** Values represent Spearman’s rank-order correlation coefficients, with * = *p* < .05, ** = ***p*** < .01, *** = ***p*** < .001. Variables reflect subscales of the Health Literacy Questionnaire (HLQ), including Understanding Health Info, Sufficient Information, Active Management, Social Support, Appraisal of Info, Engagement, Navigation, Health Literacy Ability, Ability to Take Action, and HLQ Total; the Technology Experiences and Challenges Inventory (TECHI Total); eHealth Literacy Scale (eHealth Literacy Total); and composite Privacy and Security Total scores.

Quantitative analyses revealed notable interrelationships among the health literacy domains (Fig. 3). Social Support demonstrated strong positive associations with both Active Management (*r* = .81, *p* < .001) and Engagement with Providers (*r* = .63, *p* < .001), highlighting the interconnected nature of relational and functional health literacy. eHealth literacy functioned as a bridge between traditional and digital competencies, showing moderate correlation with Technological Proficiency (*r* = .40, *p* < .01) and strong correlation with Social Support (*r* = .66, *p* < .001). Notably, Privacy and Security Concerns were not significantly correlated with any other measured variables (all *ps* > .05), suggesting that concerns about digital privacy may represent a distinct dimension of digital health engagement. Full descriptive statistics for all variables are provided in Table 3.

### Integration of Qualitative and Quantitative Analysis

**Figure 4:**
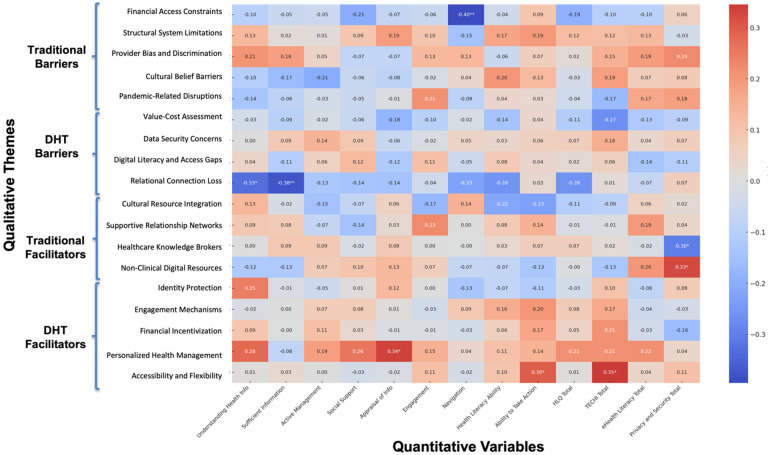
Spearman correlations between qualitative themes and quantitative measures of health literacy, technology experience, eHealth literacy, and privacy and security variables **Note.** Values represent Spearman’s rank-order correlation coefficients, with * = *p* < .05, ** = ***p*** < .01, *** = ***p*** < .001. Quantitative measures include subscales from the Health Literacy Questionnaire (HLQ; Understanding Health Info, Sufficient Information, Active Management, Social Support, Appraisal of Info, Engagement, Navigation, Health Literacy Ability, Ability to Take Action, and HLQ Total), the Technology Experiences and Challenges Inventory (TECHI Total), the eHealth Literacy Scale (eHealth Literacy Total), and composite Privacy and Security Total scores. Qualitative themes reflect sociocultural, technological, and individual-level factors identified through thematic analysis of participant interviews.

Integration enabled examination of how key qualitative themes mapped onto individual differences in health and digital competencies. Spearman’s rank-order correlations between code frequencies and quantitative measures reinforced several core qualitative insights (Fig. 4). Within traditional healthcare barriers, financial access constraints were negatively associated with healthcare navigation ability (*r* = −0.41, *p* < .01), supporting participants’ narratives that financial limitations restrict not just access but the ability to act on health information. In the DHT barrier domain, relational connection loss showed significant negative associations with understanding health information (*r* = −0.33, *p* < .05) and having sufficient information to manage health (*r* = −0.38, *p* < .01). These patterns align with participant concerns that digital platforms can dilute communication and clarity in ways that interfere with comprehension and trust.

Several correlations also differentiated how traditional and digital facilitators operate. For example, reliance on healthcare knowledge brokers (i.e., trusted individuals with medical expertise) was negatively correlated with privacy concerns (*r* = −0.30, *p* < .05), suggesting that interpersonal guidance may increase sensitivity to data security risks. In contrast, participants who used non-clinical digital resources (e.g., search engines) reported fewer privacy concerns (*r* = 0.33, *p* < .05), potentially reflecting greater comfort with decentralized information seeking. Within digital facilitators, personalized health management was positively associated with health information appraisal (*r* = 0.34, *p* < .05), consistent with participant reports that digital tools feel most empowering when users are confident in evaluating health information. Additionally, perceived accessibility and flexibility, a dominant digital facilitator, was positively associated with ability to take health-related action (*r* = 0.30, *p* < .05) and technological proficiency (*r* = 0.35, *p* < .05), reinforcing qualitative accounts of how DHTs remove logistical barriers and promote engagement.

### Social Identity Intersections

To examine how intersecting social identities shaped participants’ healthcare experiences, we used a two-pronged approach combining qualitative co-occurrence analysis and quantitative correlational analysis. These complementary methods revealed how identity-related dynamics appeared more prominently in traditional healthcare contexts, while operating in more subtle or indirect ways in digital environments.

### Co-Occurrence Analysis

Qualitative co-occurrence analysis indicated that participants referenced their social identities far more frequently when discussing traditional healthcare settings, with 94 identity-barrier/facilitator intersections coded across the transcripts. Cultural Belief Barriers were the most frequent site of identity intersection, especially with religious/spiritual identity (8 co-occurrences), race/ethnicity (6), and family roles (5). Structural System Limitations and Supportive Relationship Networks also intersected commonly with race/ethnicity, income level, and religious identity. In contrast, identity-related co-occurrences were far less common in digital healthcare narratives, with only 9 coded intersections across the dataset. Where they did appear, identity was linked with practical challenges, such as income intersecting with Affordability Concerns (3 co-occurrences) or education level intersecting with Convenience and Access (2).

### Correlational Patterns

**Figure 5:**
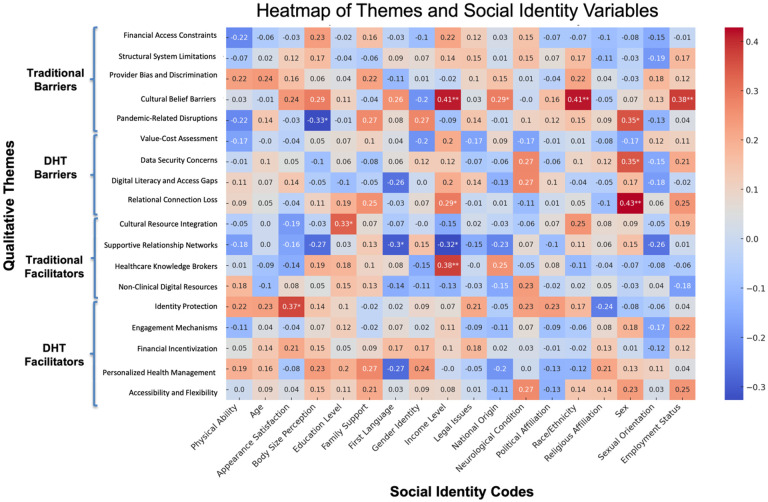
Spearman correlations between qualitative themes and participant social identity codes **Note.** Heatmap showing Spearman correlations between mentions of social identity and identified themes, with * = *p* < .05, ** = ***p***< .01, *** = ***p***< .001. Social identity codes reflect participant-reported demographic characteristics that were coded including physical ability, age, appearance satisfaction, body size perception, education level, family support, first language, gender identity, income level, legal issues, national origin, neurological condition, political affiliation, race/ethnicity, religious affiliation, sex, sexual orientation, and employment status. Qualitative themes include Traditional Barriers (e.g., financial access constraints, structural system limitations, provider bias), DHT Barriers (e.g., data security concerns, digital access gaps), Traditional Facilitators (e.g., healthcare knowledge brokers, cultural resources), and DHT Facilitators (e.g., engagement mechanisms, personalization, accessibility).

Although identity references were more commonly voiced in traditional care narratives, correlational analyses revealed measurable associations between social identity variables and specific barriers and facilitators in both traditional and digital settings (Fig. 5). In traditional healthcare, participants who referenced race/ethnicity, income, or employment status were significantly more likely also to discuss Cultural Belief Barriers (*r* = .41, *p* < .01 for both race/ethnicity and income; *r* = .38, *p* < .01 for employment status). Mentions of national origin were also positively associated with Cultural Belief Carriers (*r* = .29, *p* < .05). Participants who referenced their sexual orientation were more likely to describe Pandemic-Related Disruptions (*r* = .35, *p* < .05), while those who referenced their body size were less likely to do so (*r* = − .33, *p* < .05). Income mentions were associated with discussion of Healthcare Knowledge Brokers (*r* = .38, *p* < .01), and references to education were positively correlated with Cultural Resource Integration (*r* = .33, *p* < .05).

In digital contexts, references to participants’ social identities continued to align with distinct themes in their healthcare narratives. Participants who mentioned their sexual orientation were more likely also describe relational connection loss (*r* = .43, *p* < .01) and data security concerns (*r* = .35, *p* < .05), suggesting that identity-related considerations may shape concerns about trust, privacy, and interpersonal dynamics in digital settings. Mentions of first language and income level were negatively associated with supportive relationship networks (*r* = −.30, *p* < .05 and *r* = −.32, *p* < .05, respectively), indicating that participants who referenced these identity markers may have been more likely to report challenges in accessing meaningful digital support. Participants who mentioned income were also more likely to discuss personalized health management (*r* = .29, *p* < .05), while references to appearance satisfaction were associated with greater discussion of identity protection (*r* = .37, *p* < .05).

## Discussion

This convergent mixed-methods investigation revealed how DHTs fundamentally transform, rather than eliminate, barriers to equitable healthcare access. The finding that financial constraints persisted across traditional and digital modalities, shifting from insurance-related costs to technology-related expenses, highlights how disparities can be maintained through new forms, even as delivery models change. Rather than expanding access for underserved communities, DHTs may recreate exclusions through different mechanisms, such as device requirements, data costs, or app subscriptions. Furthermore, the emergence of novel digital-specific barriers, including relational disconnection, gaps in digital literacy and access, and privacy concerns, underscores how technological solutions can simultaneously reduce traditional obstacles while introducing new ones^37^.

This transformation also extended to facilitators. Relationship-based supports that have long been documented as critical for marginalized populations^30,31^ were replaced by engagement strategies and feedback mechanisms that often require a baseline level of digital fluency^63^. Crucially, identity-based disparities remained visible across both healthcare contexts, even as their expression shifted. While participants more frequently referenced their identities in relation to traditional healthcare, the quantitative and co-occurrence analyses revealed continued patterns in digital settings. These included associations between technological proficiency and perceived benefits of DHTs, as well as heightened privacy concerns and relational challenges among participants referencing specific identity markers such as sexual orientation and income. These findings align with broader patterns of digital inequality^24^ and suggest that digital tools may render disparities less visible, without addressing their root causes.

### Healthcare Barriers and Facilitators across Contexts

The comprehensive patterns of barriers and facilitators identified across healthcare modalities illustrate that technology does not neutralize structural inequities but rather reshapes them. Traditional barriers such as financial access constraints, provider bias, and structural limitations reflect well-documented mechanisms of exclusion, particularly for marginalized populations^10,14,15^. Cultural belief barriers, endorsed by 94% of participants, further highlight the enduring influence of intergenerational norms and community dynamics on shaping care engagement.

At the same time, the emergence of digital-specific challenges reflects new forms of exclusion. Digital literacy and access gaps (reported by 45% of participants) echo longstanding concerns about differential technology access^27,64^, while relational connection loss (endorsed by 72%) highlights how technology can disrupt the interpersonal foundations of care, especially among populations that rely heavily on trusted relationships and cultural concordance^16^. Facilitators also shifted, from culturally embedded supports and healthcare knowledge brokers to digital engagement strategies and personalization features, structures that may advantage those with more technological resources and comfort.

### Digital Transformation of Barriers and Facilitators

These shifts suggest that DHTs are not simply neutral delivery mechanisms. They restructure healthcare access in ways that can perpetuate, and at times obscure, systemic inequities. For example, while traditional financial barriers often involved insurance gaps or copays, digital contexts introduced technology costs and app-based pricing structures—forms of exclusion that may be perceived as more voluntary or personal, but are equally tied to structural inequality^28^. The apparent absence of traditional barriers, such as provider discrimination or bureaucratic inefficiencies, in digital narratives could be misinterpreted as progress. However, the asynchronous, remote, and algorithm-driven nature of DHTs may simply mask these issues rather than resolve them. This “invisibility” of disparities in digital settings may reflect reduced opportunities for discrimination to be directly observed, not its elimination. The shift from relationship-centered to technology-mediated facilitators is similarly consequential. Whereas traditional facilitators often leveraged social capital and cultural resources^30^, digital facilitators, such as those utilizing gamified features or algorithmic personalization, demand individual proficiency and sustained device engagement. The positive association between technological proficiency and perceived accessibility (*r* = .35, *p* < .05) suggests that DHTs may stratify access by digital skills, not just medical need. This has critical implications for equity; communities that previously relied on interpersonal networks may be left without comparable support in digital spaces. Without culturally grounded digital alternatives, the transition to DHTs risks privileging already-connected users while deepening divides for those with lower technological access, confidence, or trust.

### Quantitative Insights Supporting Thematic Patterns

The integration of quantitative findings further substantiates and enriches the thematic results. Correlations among health literacy domains, particularly the strong links between social support, active health management, and provider engagement (*r* = .81, *p* < .001), highlight the interconnected nature of relational and functional healthcare literacy^56^. These results reinforce the value of interpersonal support as a foundation for navigating care effectively. eHealth literacy served as a conceptual and statistical bridge between traditional and digital competencies, correlating with both technological proficiency (*r* = .45, *p* < .01) and social support (*r* = .66, *p* < .001). Similarly, higher scores on the TECHI, which measures general technological comfort and frequency of use, were positively associated with perceived accessibility and engagement with digital tools. These findings align with prior models that position eHealth literacy as essential to meaningful digital health engagement^63^. In contrast, privacy and security concerns appeared largely independent of other measures, suggesting that apprehensions about data protection form a distinct dimension of digital vulnerability that cuts across skill levels and access. Several correlations mirrored specific qualitative themes. For example, financial access constraints were negatively associated with healthcare navigation ability (*r* = −0.41, *p* < .01), reinforcing participant narratives that economic hardship affects not only whether care is pursued, but how successfully individuals engage with systems. Relational connection loss in digital contexts was associated with lower health information comprehension (*r* = −0.33, *p* < .05), highlighting how interpersonal disruptions can hinder understanding, particularly in remote formats.

### Social Identity Intersections and Digital Health Equity

Our identity-based analyses provide further insight into how DHTs may obscure, but not erase, the influence of social identity on healthcare experiences. In traditional settings, participants frequently linked identity to care barriers and facilitators, particularly around race, religion, income, and family roles^40^. These intersections reflect longstanding evidence of structural discrimination and identity-based disparities in clinical contexts^14,15^. In digital healthcare narratives, however, identity was referenced less often and in more indirect ways. This reduction in explicit identity linkage may stem from the algorithmic, asynchronous nature of DHTs, where social cues and interpersonal interactions are minimized. Yet correlational analyses revealed that identity-based disparities remained. For example, participants referencing sexual orientation reported higher relational disconnection (*r* = .43, *p* < .01) and greater data security concerns (*r* = .35, *p* < .05). Similarly, references to income or language were associated with diminished access to supportive digital networks (*r* = − .32 and − .30, respectively). These patterns align with broader concerns that digital health systems may reinforce existing disparities through seemingly neutral interfaces and algorithms^24,65^. The absence of overt discrimination does not mean equity has been achieved—it may simply mean it has become more difficult to identify and address.

### Implications for Design, Practice, and Policy

The results of this study highlight key opportunities for advancing equity in digital health by addressing the specific barriers and facilitators identified across traditional and digital contexts. First, digital health technologies should be developed using universal design principles and co-design methods that include diverse end-users to ensure cultural relevance and usability across demographic groups. This aligns with participatory design frameworks that have demonstrated success in improving health technology adoption and usability among underserved populations^66–68^. Second, hybrid care models that integrate the relational strengths of traditional care with the efficiency and scalability of digital tools may offer the best outcomes for engagement and equity. Research supports that such models can enhance care continuity, especially for populations with structural barriers to access^69,70^. Third, equity-focused evaluation metrics should be embedded in digital platforms, along with safeguards such as algorithm audits and user testing among marginalized populations to detect and mitigate bias. These strategies reflect a growing consensus on the importance of fairness and transparency in healthcare AI systems^65,71^. Finally, tailored education and outreach efforts are essential, both to raise awareness about privacy risks among low-literacy users and demystify digital tools for those with limited technological access or confidence. Such approaches are grounded in eHealth literacy models that emphasize the need for user-centered communication and digital empowerment^63,72^.

### Strengths, Limitations, and Future Directions

This study offers several notable strengths. Using a convergent mixed-methods approach, it goes beyond surface-level assumptions that digital technologies inherently increase access to care. Instead, it centers participants’ voices to examine how digital health tools transform, not simply replicate or eliminate, existing barriers and facilitators. By comparing themes across traditional and digital healthcare settings, the study identifies which barriers persist, which shift in form, and which newly emerge, offering a layered understanding of the evolving healthcare landscape. Importantly, this work foregrounds social identity as a critical factor in healthcare experiences. By examining both co-occurrence patterns in narrative data and correlations between identity mentions and other measures, the study captures how identity intersects with access and engagement. In digital health research, few studies have systematically examined how identity-based disparities translate to digital platforms, with most focusing on single-axis measures, such as broadband access or general digital literacy^24,64^. Taken together, the combination of thematic extraction, cross-context comparison, quantitative linkage, and identity-based analysis enables a uniquely nuanced and comprehensive exploration of digital health equity.

Nonetheless, several limitations warrant consideration. First, although the sample centered on historically underserved populations, findings may not generalize to other groups, such as male caregivers, non-binary individuals, or older adults, who may experience distinct healthcare dynamics. Expanding demographic representation remains an ongoing need in digital health equity research^72^. Second, data were collected during the COVID-19 pandemic, a period that rapidly reshaped healthcare delivery. While this context offered a unique lens into digital health transformation, it may have introduced temporal effects unlikely to persist post-pandemic. Longitudinal work is needed to examine whether patterns observed here hold as hybrid care models stabilize^51^.

Third, Spanish-language interviews were translated to English for analysis, which may have resulted in some loss of cultural or linguistic nuance despite professional translation services. Fourth, although our thematic coding captured whether participants mentioned aspects of their identity and the broad identity category (e.g., race, gender, sexual orientation), it did not capture the specific identity value (e.g., identifying as Black, Latina, or transgender). Coding only captured when an identity category, such as race, was referenced as relevant in participants’ narratives, not which specific group (e.g., Black, Asian, or Latine) was mentioned. Furthermore, although our approach captured explicit references to identity, it may have missed more implicit or structural forms of inequity embedded in language or digital design. Standard qualitative methods can under-detect these subtler dynamics, especially in digital contexts^73^. Future research could integrate natural language processing to detect biased or exclusionary phrasing, critical discourse analysis to examine how power and marginalization are reflected in participants’ narratives, and inclusive user experience testing to reveal how specific interface choices may unintentionally disadvantage certain groups. Together, these methods offer a more comprehensive toolkit for identifying less visible but still impactful ways bias can persist in digital healthcare systems.

## Conclusion

As healthcare continues to shift into the digital domain, this study offers a critical lens on how barriers and facilitators are not merely replaced but fundamentally transformed. Through a nuanced, mixed-methods approach, findings demonstrate that digital health tools may reduce certain logistical constraints but also introduce new challenges related to connection, privacy, and literacy, particularly for populations historically marginalized in healthcare. Rather than assuming digital tools are inherently equitable, these findings underscore the need for intentional design, inclusive evaluation, and ongoing vigilance. By centering the experiences of historically marginalized groups and integrating both qualitative and quantitative insights, we can better understand how digital transformation alters the landscape of healthcare and how to ensure it moves us toward systems that are not only innovative but also just, inclusive, and accessible.

## Supplementary Files

This is a list of supplementary files associated with this preprint. Click to download.

- TASM6.10.25.docx

## Figures and Tables

**Table 1:** Sample Demographics

Caregiver Demographic Characteristics	M *(SD)* or N *(%)*	Range
Age (year)	41.49 (6.56)	26–56
Female	47 (100%)	
Sexual Orientation		
Straight	46 (97.9%)	
Gay, Lesbian, Bisexual	1 (2.1%)	
Ethnicity		
Hispanic or Latino/a	17 (36.2%)	
Non-Hispanic or Non-Latino/a	30 (63.8%)	
Race		
American Indian/Alaska Native	1 (2.1%)	
Asian	0 (0.0%)	
Black/African American	14 (29.8%)	
Hispanic White	17 (36.2%)	
Multi-race	5 (10.6%)	
Non-Hispanic White/Caucasian	7 (14.9%)	
Other	3 (6.4%)	
Born in US	34 (72.3%)	
Education level		
Graduate degree	14 (29.8%)	
Bachelor’s degree	14 (29.8%)	
Some college but no degree	7 (14.9%)	
High school degree or equivalent (e.g. GED)	4 (8.5%)	
Associates degree	7 (14.9%)	
Less than high school degree	1(2.1%)	
Employment Status		
Full-time	27 (57.4%)	
Part-Time	5 (10.6%)	
Student	2 (4.3%)	
Homemaker	8 (17.0%)	
Currently unemployed	3 (6.4%)	
Retired	2 (4.3%)	
Annual household income before taxes		
≤$50,000 (Low)	24 (52.2%)			
$50,001–99,999(Middle)	13 (28.3%)	
≥ $100,000 (High)	9 (19.6%)	
Not reported	1 (2.12%)	
Preferred Language		
English	40 (85.1%)	
Spanish	7 (14.9%)	

**Table 2:** Theme Overview

Domain	Themes	*N/47*
Traditional Healthcare Barriers	1. Financial Access Constraints	42
	2. Structural System Limitations	37
	3. Provider Bias and Discrimination	35
	4. Cultural Belief Barriers	44
	5. Pandemic-Related Disruptions	28
DHT barriers	6. Relational Connection Loss	34
	7. Digital Literacy and Access Gaps	21
	8. Data Security Concerns	17
	9. Value-Cost Assessment	13
Traditional Healthcare Facilitators	10. Supportive Relationship Networks	44
	11. Healthcare Knowledge Brokers	36
	12. Cultural Resource Integration	26
	13. Non-Clinical Digital Resources	11
DHT Facilitators	14. Accessibility and Flexibility	45
	15. Personalized Health Management	27
	16. Financial Incentivization	11
	17. Engagement Mechanisms	19
	18. Identity Protection	3

**Note.** *N* refers to the number of individuals who endorsed the theme.

**Table 3:** Descriptive Statistics for Quantitative Variables

Measures	M *(SD)*	Range
Health Literacy Scale Total	158.13 (20.00)	115–197
‘Feeling understood and supported by healthcare providers’	13.23 (3.01)	4–16
‘Having sufficient information to manage health’	12.37 (2.47)	8–16
‘Actively managing health’	14.53 (2.80)	8–20
‘Social Support for health’	15.86 (2.67)	11–20
‘Appraisal of health information’	14.81 (2.74)	8–20
‘Ability to actively engage with healthcare providers’	20.83 (3.66)	9–25
‘Navigating the healthcare system’	24.09 (4.03)	16–30
‘Ability to find good health information’	19.55 (4.54)	10–25
‘Understanding health information well enough to know what to do’	22.87 (2.35)	18–25
e-Health Literacy Scale	33.79 (5.15)	19–40
Attitudes towards Mobile Privacy and Security	8.85 (2.47)	3–13
Technological Ease and Computer Based Habits Inventory	65.40 (10.10)	47–85

*Note. M* = mean; *SD* = standard deviation, range indicates minimum to maximum scores.

**Table 4.** Overview of Theme and Social Identity Code Cooccurrence

	Social Identity Codes																	
Themes	BS	ND	I	AP	FE	WE	PB	F	LS	RS	MP	SO	FL	NO	GI	RE	A	SUM
Trad Barriers																		
Financial	1	1	1	1	1	1	1	1	1	1	1	1	1	1	1	1	1	17
Structural	2	0	1	0	0	1	1	1	2	3	0	0	0	1	0	8	0	20
Bias/Discrim	0	0	0	0	0	0	0	0	0	0	0	0	0	0	0	0	0	0
Cultural/	0	1	1	0	2	1	0	5	1	8	1	0	5	6	0	21	1	53
Pandemic	0	0	0	0	2	0	0	1	0	1	1	0	0	0	0	0	0	5
Sum	3	1	5	0	4	7	2	8	4	12	3	0	5	8	0	31	1	94
Trad Facilitators																		
Relationships	1	0	0	0	3	4	0	7	0	1	1	0	0	3	0	2	2	24
Medical Proxy	0	0	3	0	2	3	0	1	0	1	0	0	2	0	0	0	0	12
Cultural Values	0	0	0	0	1	0	0	0	1	13	1	0	2	5	1	13	0	37
Non-DHT	0	0	0	0	0	0	0	0	0	0	0	0	0	0	0	0	0	0
Sum	1	0	3	0	6	7	0	8	1	15	2	0	4	8	1	15	2	73
DHT Barriers																		
Relational	0	0	0	0	1	0	0	0	0	0	0	0	0	0	0	0	0	1
Tech/Access	0	0	0	0	0	0	0	0	0	0	0	0	0	0	0	0	0	0
Data security	0	0	1	0	0	0	0	0	0	1	0	0	0	0	0	0	0	2
Cost/Value	1	0	1	0	0	0	0	0	0	1	0	0	0	0	0	0	0	3
Sum	1	0	1	0	1	0	0	0	0	1	0	0	0	0	0	0	0	5
DHT Facilitators																		
Conv/Access	0	0	0	0	0	2	0	0	0	0	0	0	0	0	0	0	0	2
Personalization	0	0	0	0	0	0	0	0	0	0	0	0	0	0	0	0	0	0
Cost Efficiency	0	0	1	0	0	0	0	0	0	0	0	0	0	0	0	0	0	1
Engag/motivation	0	0	0	0	0	1	0	0	0	0	0	0	0	0	0	0	0	1
Anon/privacy	0	0	0	0	0	0	0	0	0	0	0	0	0	0	0	0	0	0
Sum	0	0	1	0	0	2	0	0	0	0	0	0	0	0	0	0	0	4

*Note*. Denotes the number of times specific social identity codes emerged with the specific themes. Body= Body size; ND = Neurodiversity; I = Income; AP= Appearance; FE = Formal education; WE = Work experience; PB= Political belief; F = Family; LS = Legal status; RS= Religious/spirituality; MP= Mental/physical ability; SO = Sexual orientation; FL =First language; NO= National Origin, GO= Gender Identity; RE= Race or Ethnicity; A= Age; Sum indicates total codes in column/row

## Data Availability

The datasets generated and analyzed during the current study are not publicly available due to privacy and confidentiality restrictions for human participants, but are available from the corresponding author on reasonable request and with appropriate ethical approvals. The datasets generated and analyzed during the current study are not publicly available due to privacy and confidentiality restrictions for human participants as specified in the IRB approval and informed consent agreements. Qualified researchers may request access to de-identified quantitative data from the corresponding author with appropriate ethical approvals and data use agreements. Qualitative interview data cannot be shared due to the risk of participant re-identification despite de-identification efforts.
